# Exploiting metabolic vulnerabilities for personalized therapy in acute myeloid leukemia

**DOI:** 10.1186/s12915-019-0670-4

**Published:** 2019-07-18

**Authors:** Lucille Stuani, Marie Sabatier, Jean-Emmanuel Sarry

**Affiliations:** Centre de Recherches en Cancérologie de Toulouse, UMR1037, Inserm, Université de Toulouse 3 Paul Sabatier, Equipe Labellisée LIGUE 2018, F-31037 Toulouse, France

## Abstract

Changes in cell metabolism and metabolic adaptation are hallmark features of many cancers, including leukemia, that support biological processes involved into tumor initiation, growth, and response to therapeutics. The discovery of mutations in key metabolic enzymes has highlighted the importance of metabolism in cancer biology and how these changes might constitute an Achilles heel for cancer treatment. In this Review, we discuss the role of metabolic and mitochondrial pathways dysregulated in acute myeloid leukemia, and the potential of therapeutic intervention targeting these metabolic dependencies on the proliferation, differentiation, stem cell function and cell survival to improve patient stratification and outcomes.

Acute myeloid leukemia (AML) is a heterogeneous group of hematological malignancies and represents the most frequent cause of leukemia-related deaths [1]. It arises from genetic abnormalities in hematopoietic stem or progenitor cells, inducing uncontrolled growth and an accumulation of abnormal myeloblasts, leading to bone marrow failure and often death. For the past three decades, standard intensive induction therapy involved a combination of cytarabine plus anthracycline cytotoxic chemotherapy. Despite a high rate (70–80%) of complete remission after standard front-line chemotherapy, the prognosis remains poor, especially for older patients. This mainly results from the high frequency of distant relapses caused by tumor regrowth initiated by chemoresistant leukemic clones after chemotherapy [2, 3]. Therefore, more specific and safe therapeutics are urgently needed. One area of high interest and potential is targeting metabolic and mitochondrial pathways that are important in AML biology and that may constitute an Achilles heel of AML cells. This review focuses on metabolic pathways dysregulated in AML, and especially in several cytogenetically defined patient subgroups, and how targeting these metabolic dependencies impacts proliferation and cell survival in this disease.

## Major metabolic dysregulations in acute myeloid leukemia

Metabolism is altered in most, if not all, cancer cells, regardless of the tumor type [4]. A key alteration in cancer metabolism is the increase in glucose uptake required to satisfy energetic and anabolic demands. It is now well established that the metabolic reprogramming undergone by transformed cells extends far beyond glycolysis and the Warburg effect, and changes in cell metabolism have fundamental implications for tumor biology and therapy [5, 6].

### Glucose metabolism

**Fig. 1:**
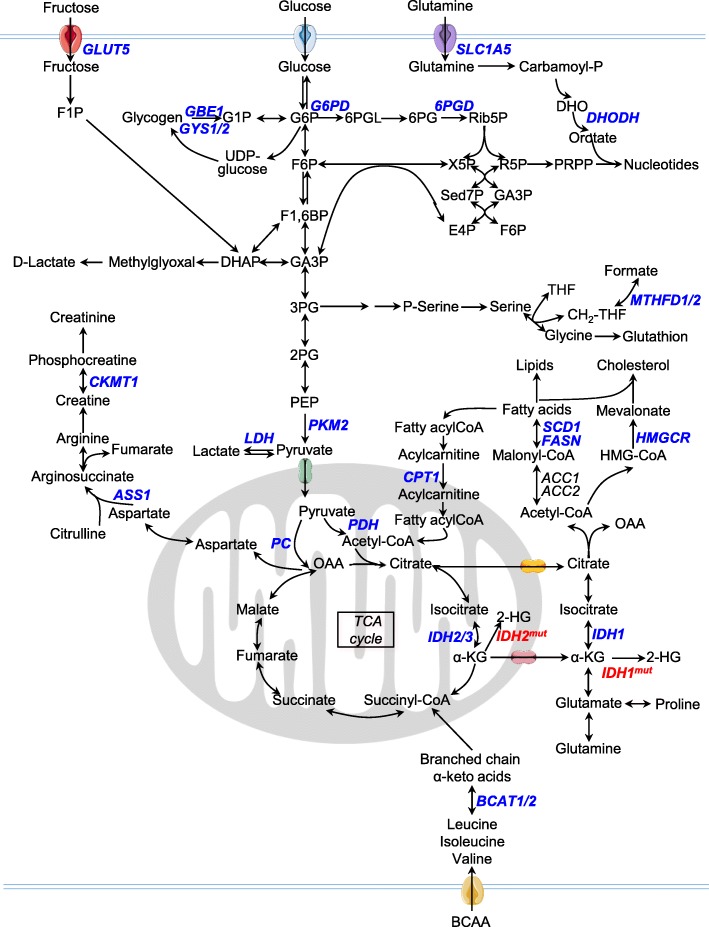
Metabolic pathways relative to deregulated reactions in myeloid leukemia. Enzymes discussed in this review are in *blue*. Compound abbreviations: *F1P* fructose-1-phosphate, *G1P* glucose-1-phosphate, *G6P* glucose-6-phosphate, *F6P* fructose-6-phosphate, *F1,6BP* fructose-1,6-biphosphate, *GA3P* glyceraldehyde 3-phosphate, *DHAP* dihydroxyacetone phosphate, *3PG* 3-phosphoglycerate, *P-Serine* phosphoserine, *2PG* 2-phosphoglycerate, *PEP* phosphoenolpyruvate, *6PGL* 6-phosphogluconolactone, *6PG* 6-phosphogluconic acid, *Rib5P* ribulose-5-phosphate, *X5P* xylulose-5-phosphate, *R5P* ribose-5-phosphate, *Sed7P* sedoheptulose-7-phosphate, *E4P* erythrose-4-phosphate, *PRPP* phosphoribosyl pyrophosphate, *Carbamoyl-P* carbamoyl phosphate, *DHO* dihydroorotate, *THF* tetrahydrofolate, *OAA* oxaloacetate, *α-KG* α-ketoglutarate, *2-HG* 2-hydroxyglutarate, *BCAA* branched-chain amino acid

Higher aerobic glycolysis in cancer cells, reported almost one century ago by Otto Warburg and known as the Warburg effect [7, 8], has sparked debate over the role of glycolysis and oxidative phosphorylation in normal and cancer cells. Since Warburg’s discovery and especially during the past 20 years, considerable efforts have been made to better understand glucose utilization in cancer cells, in particular to determine if inhibiting glycolysis or other glucose-dependent pathways could represent promising therapeutic approaches. It has been suggested that AML patients exhibit a high glycolytic metabolism at diagnosis that is potentially associated with favorable outcomes [9], even if the number of patients in this study remains small. Another study reported that a six-metabolite signature (including pyruvate and lactate) related to the crosstalk between glycolysis and mitochondria was specifically enriched in the serum of patients at diagnosis compared to healthy controls and demonstrated prognostic value in cytogenetically normal AML (CN-AML) patients as it could predict poor survival for these patients [10]. Interestingly, deletions of the two glycolytic enzymes PKM2 and LDHA, which catalyze the production of cytosolic pyruvate and lactate, respectively, inhibit leukemia initiation in vivo in AML mice models while preserving normal hematopoietic stem cell function [11] (Fig. 1).

Glucose metabolism is also involved in other crucial metabolic pathways such as the pentose phosphate pathway (PPP) coupled to NADPH production, glutathione/redox recycling, and nucleotide biosynthesis (Fig. 1). Overexpression of glucose-6-phosphate dehydrogenase (G6PD) has been reported to correlate with an adverse prognosis in an AML cohort [12]. Moreover, in vitro and in vivo inhibition of 6-phosphogluconate dehydrogenase (6PGD) and G6PD demonstrated anti-leukemic activities and synergized with cytarabine [12–15]. Inhibition of 6PGD leads to impaired lipogenesis through reactivation of LKB1-AMPK signaling [14]. Sensitivity to G6PD inhibition is driven by mTORC1 activity as mTORC1 activation leads to glucose addiction in AML. Inhibition of mTORC1 induces a switch toward oxidative metabolism and survival of AML cells [12]. Furthermore, the anti-leukemic effects of mTOR inhibitors are enhanced when combined with anti-glycolytic agents, underscoring the strong interconnection between mTOR activity and leukemic metabolism [16]. Better characterization of mTOR-associated metabolic alterations would help in the design of new combinatory therapeutic approaches and/or help distinguish patients who could better benefit from these treatments. This will be even more important since no clear evidence of clinical efficacy has been found by several clinical trials of agents targeting mTOR kinase in myeloid leukemia [17–22] (Table 1). This modest efficacy is due to multifactorial aspects of mTOR biology and AML heterogeneity. The anti-leukemic effect of mTOR inhibition depends on the level of constitutive PI3K/Akt/mTOR pathway activation, leukemia-microenvironment crosstalk, and the release of mediators by both AML and stromal cells [71].

**Table 1:** Drugs targeting metabolic activities in myeloid leukemia

Target protein or process	Pathway impacted	Drug	Patient group	Preclinical studies	Clinical trials
Hexokinases	Glycolysis	2-Deoxyglucose	AML	[10]	**-**
			AML with FTL3-ITD mutation	[23–25]	**-**
mTOR kinase	mTOR-dependent metabolic pathways	Sirolimus (rapamycin), temsirolimus, everolimus	AML/ CML	[19–22]	Phase I/II
Glutaminase	Glutaminolysis	CB839	AML	[26, 27]	Phase I
			AML with IDH mutations	[28]	-
Asparagine glutamine availability	Amino acid metabolism	Erwinase alone L-asparaginase (encapsulated in red blood cells) + low-dose cytarabine	AML		Phase I/II
Arginine availability	Nucleotides polyamines biosynthesis	ADI-PEG20	AML	[29]	Phase I/II [30]
CKMT1	Creatine biosynthesis and OxPHOS	Cyclocreatine	AML with EVI1 aberrant expression	[31]	-
Mitochondrial protein translation	OxPHOS	Tigecycline	AML	[32]	Phase I
Mitochondrial protease ClpP	OxPHOS	A2-32-01	AML	[33]	-
mtDNA polymerase	OxPHOS	2'3'-Dideoxycytidine	AML	[34]	-
ETC complex I	OxPHOS	Metformin	AML	[35, 36]	Phase I
		IACS-010759	AML	[37]	Phase I
DHODH	Nucleotides and OxPHOS	Brequinar sodium BRQ	AML	[38]	Phase I/II
		HZ00	CML	[39]	-
		Isobavalchone	AML	[40]	-
		PTC299	AML	[41]	Phase Ib
CPT1a	Fatty acid oxidation	Etomoxir	AML	[42, 43]	-
		Avocatin B		[44, 45]	-
		ST1326		[46]	-
Mitochondrial anti-apoptotic BCL2	OxPHOS and pyrimidine biosynthesis	Venetoclax ABT-199	AML/ CML	[47–49]	Phase I/II/III [50]
			AML with FTL3-ITD mutation	[51]	Phase I/II in combination with FLT3-ITD inhibitor
			AML with IDH1 mutation	[52]	Phase I/II in combination with IDH1 mutant inhibitor
Amino acid transporters	AA metabolism and OxPHOS	Venetoclax ABT-199 + azacitidine	AML	[48]	Phase I/II/III [50]
IDH2 mutant enzyme	2-HG production	Enasidenib AG-221	AML with IDH2 mutation	[53–55]	FDA approved phase I/III
IDH1 mutant enzyme	2-HG production	Ivosidenib AG-120	AML with IDH1 mutation	[56, 57]	FDA approved phase I/III
		BAY1436032		[58–60]	Phase I
		IDH305			Phase I [61]
IDH1/IDH2 mutant enzyme	2-HG production	Vorasidenib AG-881	AML with IDH1 and/or IDH2 mutation		Phase I [62]
HMG-CoA reductase	Mevalonate biosynthesis	Statins: lovastatin, pravastatin	AML	[63–65]	Phase I/II [66, 67]
Stearoyl CoA desaturase 1	Lipid biosynthesis	BaP = combination of lipid-regulating bezafibrate and the sex hormone medroxyprogesterone acetate	AML	[68–70]	-

### Amino acid metabolism

Of note, Willems et al. have shown that glutamine availability is a limiting step for mTORC1 activation and that the anti-tumor effect of L-asparaginase is mainly due to its glutaminase activity in AML [72], highlighting a major role for amino acids in leukemia biology. Indeed, intracellular glutamine concentration controls the uptake of leucine as leucine is imported into the cell in exchange for glutamine by the SLC7A5/3A2 transporter and leucine is required for Rheb-mediated mTOR activation at the lysosomal surface [73, 74]. Glutamine is a non-essential amino acid and one of the major carbon sources used by cancer cells for proliferation in vitro [75, 76]. It is also an important nitrogen donor for amino acids and nucleotides and a major substrate for TCA cycle intermediates as well as glutamate and aspartate [77–79] (Fig. 1). Dependence of leukemic cells on glutamine for tumor growth has been reported, and knockdown of the glutamine transporter SLC1A5 abrogates tumor development in mice [72].

An approach to extend therapeutic opportunities beyond glycolysis and glutaminolysis may be found in the identification of auxotrophic amino acids required by AML cells. It has been reported that most AML patients are deficient in arginosuccinate synthetase-1 (ASS1), an enzyme that allows the conversion of citrulline and aspartate into the arginine precursor argininosuccinate [29] (Fig. 1). The loss of ASS1 has been reported in other tumor types where it is required to support cell proliferation and nucleotide synthesis by sustaining the intracellular aspartate level [80]. A decrease in ASS1 can also lead to a dependence on arginine, which has been explored as a potential vulnerability in different cancer types, including AML [29].

### Lipid and sterol metabolism

De novo lipid biosynthesis is another metabolic pathway highly reprogrammed in cancer and leukemic cells, in particular to increase biomass. Numerous studies support targeting lipid synthesis for therapeutic benefit [81, 82]. Inhibition of key lipogenic enzymes, fatty acid synthase (FASN) [83] and stearoyl CoA desaturase 1 (SCD1) [68], have been shown to disrupt lipid synthesis and induce apoptosis in AML (Fig. 1). SCD1 inhibition was obtained through treatment with BaP, a combination of lipid-regulating bezafibrate and the sex hormone medroxyprogesterone acetate [68] (Table 1). BaP disrupts prostaglandin metabolism, leading to AML growth arrest and differentiation [68–70]. Interestingly, it was reported that BaP treatment caused redirection of pyruvate utilization leading to conversion of α-ketoglutarate (α-KG) to succinate and of oxaloacetate into malonate to cope with oxidative stress [68, 84–86]. This pyruvate reprogramming by BaP includes preferential activation of pyruvate carboxylase (PC) over pyruvate dehydrogenase (PDH) to produce malonate, a competitive inhibitor of the succinate dehydrogenase [87–89] (Fig. 1). PC has been shown to play a key role in different solid tumors, in particular through in vivo reprogramming of glucose utilization to support anapleurosis [90–95]. Further investigations of PC activity in leukemia, especially in vivo, would be highly valuable and provide a better understanding of pyruvate metabolism and channeling between glycolysis, TCA cycle, and amino acid pathways.

**Fig. 2:**
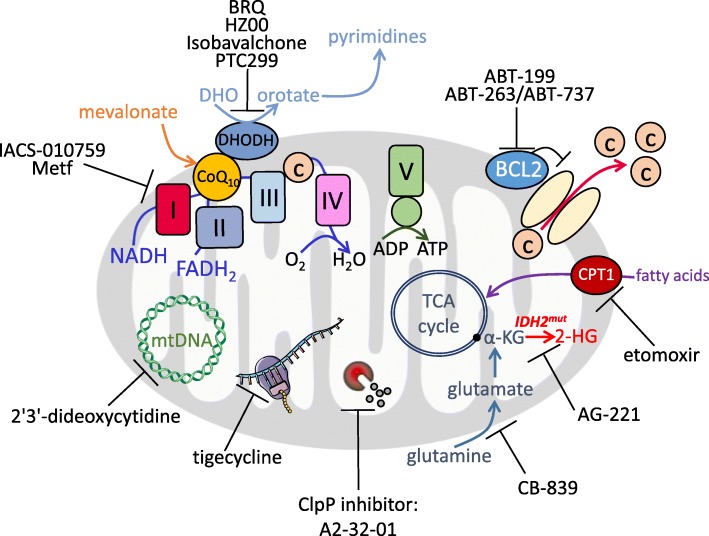
Pharmacological inhibitors used to disrupt mitochondrial activities in myeloid leukemia

Various studies have focused on the mevalonate pathway and the inhibition of the rate-limiting enzyme 3-hydroxy-3-methylglutaryl-coenzyme A (HMG-CoA) with statins in AML [63, 96] (Fig. 1). The end-products of the mevalonate pathway include cholesterol, a major constituent of cell membranes, but also ubiquinone, which is involved in electron transfer between the Electron transfer chain (ETC) complexes I to III (see below; Fig. 2), geranylgeranyl and farnesyl pyrophosphate, which are necessary for post-translational modification of oncogenic proteins, and tyrosine kinase (TK) receptors [97].

### Oxidative phosphorylation and mitochondrial metabolism

Originally, observations by Otto Warburg that cancer cells exhibited higher glycolytic activity than normal cells even in the presence of oxygen led to the assumption that cancer cell mitochondrial respiration may be impaired. Since then, major studies have clearly demonstrated that cancer cells are able to use oxygen via oxidative phosphorylation (OxPHOS) [98–101] and mitochondria are essential for cancer cell survival. In myeloid leukemia, Ṧkrtić et al. observed that AML cells had higher mitochondrial mass and an increased oxygen consumption rate compared to normal hematopoietic progenitors [32]. Of note, bulk cell populations had higher mitochondrial mass than an immature CD34^+^CD38^−^ cell population, suggesting unique mitochondrial characteristics of leukemic stem cells (LSCs). However, the increased mitochondrial mass in AML did not translate into an increase in ETC complex I, III, IV, and V activities, resulting in a lower capability of AML compared to normal cells to enhance their maximal respiration with higher electron flux, known as the spare reserve capacity, suggesting a decreased ability to cope with oxidative stress [102]. In addition, different studies have reported an amplification of mitochondrial DNA (mtDNA) levels in AML [34, 103] that correlates with enhanced cytoplasmic nucleoside kinase expression [34, 104]. Almost 20 years ago, Beuneu et al. reported that dihydro-orotate dehydrogenase (DHODH), a mitochondrial enzyme of de novo pyrimidine biosynthesis that catalyzes the ubiquinone-mediated conversion of dihydro-orotate (DHO) to orotate, could provide electrons to the ETC via ubiquinone in AML cells [105]. Therefore, inhibition of DHODH could represent another promising approach to tackle mitochondria in cancer.

Fatty acids can be a major source for TCA cycle precursors and mitochondrial respiration, especially during and following metabolic challenges or limitations of other oxidizable substrates [82, 106] (Fig. 2). Increased fatty acid oxidation (FAO) and high carnitine palmitoyltransferase 1 (CPT1a) expression have been associated with a poor prognosis in normal karyotype AML patients [107, 108]. German et al. [109] observed a key role of prolyl-hydroxylase 3 (PHD3) in FAO regulation in AML. They reported that, in the setting of high nutrient abundance, PHD3 activates acetyl-CoA carboxylase 2 (ACC2) via hydroxylation, causing inhibition of CPT1a and FAO. Accordingly, when nutrients are scarce and energetic stress is induced, AMPK phosphorylates and inhibits ACC2 to activate FAO [110, 111]. Reduced expression of PHD3 could therefore represent a marker of good responders to FAO inhibitors in AML.

## Targeting metabolic vulnerabilities in acute myeloid leukemia

As metabolic alterations are part of oncogenesis and tumor progression, cancer cell metabolism offers promising targets for therapeutic intervention. Hereafter, we discuss several key metabolic pathways that might be therapeutically targetable for AML treatment.

### Tackling aerobic glycolysis

Treatment with 2-deoxyglucose (2-DG) to inhibit aerobic glycolysis and related glycosylation of oncogenic proteins exerts an anti-proliferative effect in different AML cell lines and patients and synergizes with conventional cytarabine chemotherapy [10, 23]. However, targeting aerobic glycolysis has not shown great success in clinical settings as 2-DG treatment necessitates high dosing that might induce hypoglycemia and cardiac and red blood cell toxicities due to PPP alteration. Moreover, LDH inhibitors have never progressed into clinical trials (Table 1). Another way to approach high glycolytic metabolism in myeloid leukemia could be through direct targeting of the glucose storage pathway or inhibition of other glycolytic sources such as glycogen and fructose (Fig. 1). It is notable that mRNA levels of glycogen biosynthetic enzymes GYS1/2 and GBE1 were associated with poor survival in AML and that invalidation of GYS1 delayed tumor growth in vivo [112]. AML cells may additionally rely on fructose under low glucose conditions through upregulation of the GLUT5 transporter to maintain glycolytic flux and overcome glucose restriction. Expression of SLC25A5, which encodes GLUT5, is associated with poor AML patient outcome and pharmacological inhibition of GLUT5 eliminates leukemic phenotypes and potentiates the effect of cytarabine in vivo [113].

### Glutaminolysis inhibition and amino acid depletion

Targeting glutaminolysis has been investigated as a promising therapeutic target in myeloid leukemia [26, 114, 115]. Of particular interest, inhibition of glutaminase with CB-839 reduces mitochondrial activities and TCA cycle intermediate levels, suggesting that glutamine exerts control on mitochondrial oxidative metabolism in AML [26, 116] (Fig. 2). Clinical trials are currently in progress to assess the benefit of the allosteric glutaminase inhibitor CB-839 (Table 1) with mixed evidence of clinical efficacy. Willems et al. have shown that the ability of L-asparaginase (kidrolase and erwinase) to transform extracellular glutamine into glutamate leads to inhibition of mTORC1 and protein translation in AML cells and that L-asparaginase exhibits anti-leukemic activities [72]. L-asparaginase, which mainly catalyzes the hydrolysis of L-asparagine to L-aspartic acid, is one of the standard drugs for treatment of acute lymphoblastic leukemia (ALL). These patients lack L-asparagine synthetase (ASNS), the enzyme that catalyzes the biosynthesis of L-asparagine, leading to a higher dependency on this amino acid [117]. However, AML patients harbor variable expression of ASNS that could explain their reduced sensitivity to L-asparaginase. Another recent study proposed another explanation linked to the bone marrow microenvironment [118]. The study by Michelozzi et al. suggests that while AML cells are sensitive to L-asparaginase, mesenchymal stromal cells (MSCs) and monocytes/macrophages produce lysosomal cysteine protease cathepsin B able to inactivate L-asparaginase. This contribution of the bone marrow microenvironment to asparaginase resistance was also described in ALL through release of asparagine and glutamine by adipocytes [119].

Depletion of arginine using a mycoplasma-derived enzyme of arginine deiminase formulated with polyethylene glycol (ADI-PEG20) that degrades arginine to citrulline reduces tumor burden in AML and synergizes with cytarabine in vitro and in vivo. Normal human hematopoietic stem-progenitor cells express higher ASS1 than AML cells, supporting the idea of selective targeting of leukemia cells and highlighting a potential therapeutic window for ADI-PEG20 [29, 30], currently under phase 2 clinical evaluation (Table 1).

### Inhibition of the mevalonate pathway

The anti-leukemic effects of statins, HMG-CoA inhibitors, have been studied [96, 120] and found to be additive with conventional chemotherapies such as cytarabine and daunorubicin in primary AML samples compared to healthy donors [63–65]. Phase I and then phase II clinical trials combining pravastatin with idarubicin and cytarabine for relapse cases of AML have shown an encouraging response rate of 75% [66, 67] (Table 1). However, a subsequent investigation of this regimen has not confirmed these encouraging results in patients with newly diagnosed AML or MDS [121]. These differences between response in newly diagnosed AML or patients at relapse could be due to rewiring of intracellular cholesterol metabolism and sterol membrane transport following chemotherapy and suggest that statins could play a role in overcoming chemoresistance rather than synergizing with frontline therapies. The focus of this review does not include deciphering all the adaptive mechanisms induced by chemotherapeutic agents or new drugs in AML, although this is important for understanding the clinical relevance of these metabolic inhibitors.

### Hitting at OxPHOS, BCL2, and mitochondrial dependencies

Mitochondria are dynamic organelles that play a crucial role in several fundamental signaling and metabolic processes such as reactive oxygen species (ROS) regulation, energy production, calcium signaling, TCA cycle, and pyrimidine or heme biosynthesis. Mitochondrial metabolism represents a targetable vulnerability due to the enhanced dependency on mitochondrial energetics of AML cells. Various strategies to disable mitochondrial function have been investigated in myeloid leukemia, including inhibition of mitochondrial translation with tigecycline [32], inhibition of the mitochondrial protease ClpP, thereby decreasing ETC complex II activity [33], and inhibition of mtDNA polymerase using 2’,3’-dideoxycytidine, a drug already used in the treatment of AIDS [34] (Fig. 2; Table 1). Each of these treatments had anti-leukemic properties in vitro and in vivo. Moreover, we and other investigators have shown that metformin, a common biguanide used to treat type 2 diabetes, exhibits anti-leukemic activities in AML [35, 36] (Table 1). However, metformin pharmacokinetics and its maximum efficient dose do not allow its use as an anti-AML agent alone in a clinical setting. Nevertheless, metformin (or other biguanides) might be promising in combination with chemotherapies or other targeted therapies, as recently shown in diffuse large B cell lymphoma refractory to all anti-CD20-based therapies using L-asparaginase, mTOR inhibitor, and metformin (called KTM therapy) [122]. Whereas metformin inhibits ETC complex I activity and thus mitochondrial oxygen consumption, high basal glucose consumption and Akt levels can also affect metformin sensitivity, suggesting combinatory therapies with AKT inhibitors may be effective [35]. More recently, the new ETC complex I inhibitor IACS-010759, which inhibits OxPHOS and nucleotide biosynthesis by decreasing aspartate levels [37], is in a phase I clinical trial for AML and solid tumors [37] (Fig. 2; Table 1).

FAO is a key catabolic pathway involved in the generation of NADH and FADH_2_, which are the electron donors of complex I and complex II of the ETC, respectively, and leading to the production of acetyl-CoA. This latter plays a crucial role in energy generation, biosynthesis, and epigenetic control through post-translational protein modifications. Inhibiting FAO has been investigated in myeloid leukemia [42, 44, 46, 114, 123]. Inhibition of CPT1a, which catalyzes the transfer of the acyl group from fatty acyl CoA to carnitine and constitutes the rate limiting step of FAO, with the aminocarnitine derivative ST1326 [45, 46], lipid Avocatin B [44], or etomoxir [42, 43] has shown anti-leukemic properties (Table 1).

As an inner mitochondrial membrane protein associated with the ETC, DHODH links de novo pyrimidine biosynthesis to mitochondrial bioenergetics. In this context, Sykes et al. [38] found that its inhibition with brequinar sodium (BRQ) abrogates the myeloid differentiation blockade and leads to anti-leukemic activities in a diverse range of AML subtypes. This can be rescued by addition of extracellular uridine. Very recently, two other newly developed DHODH inhibitors for AML and one for chronic myeloid leukemia (CML) have been described [39–41] (Fig. 2; Table 1). Although BRQ has not shown benefits in early phase clinical trials with solid cancers, it has not yet been studied in hematological malignancies [124–128]. Because BRQ has been shown to lead to a potent induction of myeloid differentiation and decrease leukemic burden, the role of DHODH in AML metabolism merits further study.

Another very exciting approach to trigger mitochondrial priming of cell death is through treatment with anti-apoptotic BCL2 inhibitors [52, 129] (Fig. 2). Lagadinou et al. demonstrated that LSCs are characterized by low levels of ROS. These ROS-low LSCs are dependent on OxPHOS via amino acid uptake for respiration rather than glycolysis and overexpress BCL2 anti-apoptotic proteins [47, 48]. Thus, pharmacological inhibition of BCL2 with the drug ABT-199 (venetoclax) impairs mitochondrial respiration and selectively targets ROS-low LSCs unable to switch to glycolysis/glucose or FAO to maintain energy production [47]. Clinical trials with venetoclax monotherapy in relapsed/refractory AML have shown a very low response rate due to a lack of apoptosis induction while mitochondrial priming is activated by this treatment to induce cell death. However, results from a phase 1b study in elderly patients with previously untreated AML on venetoclax treatment in combination with hypomethylating agents (azacitidine and decitabine) reported a 61% overall response [50] (Table 1). Treatment with venetoclax plus azacitidine inhibited amino acid uptake and induced disruption of the TCA cycle, inhibition of ETC complex II, and impairment of OxPHOS in ROS-low LSCs [48, 49]. Pharmacological inhibition of amino acid metabolism also decreased OxPHOS and induced cell death in AML [48] (Table 1). Previous work has suggested that FAO could be involved in BCL2 regulation and BAX- and BAK-dependent mitochondrial permeability transition pore formation through interactions between CPT1 and the pro-apoptotic BH3-only protein Bid [130] or BCL2 [131], highlighting a dual interest in FAO inhibition and synergy with BH3 mimetics in AML. Because many of the reported manipulations of metabolic pathways have been shown to modulate BCL2 expression or dependence, combinations of metabolic inhibitors and BCL2 inhibitors are of special interest. For example, statins also enhanced ABT-199 efficacy in AML through the inhibition of protein geranyl-geranylation, which leads to BCL2 modulation and upregulation of pro-apoptotic BH3 only proteins PUMA [132] and etomoxir, increasing the therapeutic efficacy of ABT-737 in vivo [43]. Very recently, a study has elegantly mapped metabolic pathways that are specifically implicated in ABT-199-induced apoptotic cell death, and demonstrated that the heme biosynthetic pathway is the major regulator of mitochondrial priming of apoptosis through ETC and OxPHOS in AML [133]. Altogether, these studies strengthen the scientific rationale for clinical development of new combinations of venetoclax and OxPHOS (or FAO) inhibitors (Table 1).

## Metabolic stratification to decipher specific vulnerabilities and develop more efficient therapies in patient genetic subgroups

For diagnosis and management of AML, a prognostic stratification has been proposed based on criteria for progressive disease and for the genomic landscape of the disease [134]. However, metabolic features have not been taken into consideration yet. As more and more studies are highlighting metabolic specificities driven by mutations in AML and as specific inhibitors of some of these mutations are displaying very promising results in clinical trials, investigating the link between genetic stratification, metabolic dependencies, and response to these specific inhibitors is particularly important. This may be crucial in order to propose better combinations of these new drugs, understand mechanisms of resistance to them, and potentially identify early markers of response.

### Isocitrate dehydrogenase mutations

In 2009, recurrent mutations in genes of two crucial metabolic enzymes, cytosolic isocitrate dehydrogenase (IDH)1 and mitochondrial IDH2, were observed in about 20% of AML patients [135–138], reinforcing the importance of furthering metabolic investigations in AML. While wild-type IDH (IDH WT) catalyzes the conversion of isocitrate to α-KG and generates NADPH, mutant IDH catalyzes a neomorphic enzyme activity that oxidizes NADPH and produces the oncometabolite 2-hydroxyglutarate (2-HG) from α-KG [139, 140]. The impact of monoallelic IDH mutation and the related accumulation of 2-HG have been well documented, in particular its effect on α-KG-dependent dioxygenase activity and subsequent effects on numerous cellular functions in these cancers, such as alteration of DNA and histone methylation and biased myeloid/erythroid differentiation [141–152].

Beyond epigenetic modifications and chromatin remodeling, 2-HG has multi-faceted roles in AML biology and leukemic transformation by competitively inhibiting multiple classes of αKG-dependent dioxygenases involved in metabolic reprogramming, BCL2-dependent cell survival, and cellular defense against oxidative stress. As IDH mutations are early events in oncogenesis and are systematically conserved at relapse [153, 154], IDH1/2 mutated enzymes represent attractive therapeutic targets [53, 144, 155–157] and small molecules selectively inhibiting the mutated forms of these enzymes have been developed and very recently approved for clinical studies [54, 56, 58–62] (Table 1). Both the IDH2m- and IDH1m-specific inhibitors promote differentiation and reduce methylation levels as well as significantly decreasing 2-HG levels [53, 54, 57, 157, 158]. However, while clinical trials are highly encouraging (up to 40% overall response rate in monotherapy in phase I/II for relapsed or refractory AML patients), resistance is routinely observed [54–57, 159].

Moreover, suppression of serum 2-HG levels alone did not predict response in AML patients, as non-responders also displayed a significant decrease in the amount of 2-HG [54, 55, 57, 160, 161]. Thus, targeting IDH mutant activity alone is not sufficient to achieve a durable clinical response in relapsed AML and new combinatory approaches need to be designed. Given the crucial roles of wild type IDH1/2 in cell metabolism (e.g. Krebs cycle, OxPHOS, cytosolic and mitochondrial redox, anabolism including lipid biosynthesis), a better understanding of the contribution of oncogenic IDH mutations to AML cell intermediary metabolism and α-KG homeostasis is expected to lead to new therapeutic strategies.

Because α-KG is the direct precursor of 2-HG, various studies have investigated the glutaminolysis pathway in IDH mutant cells and reported that glutamine was indeed the main source of 2-HG production [139, 162]. Therefore, inhibition of glutaminolysis with different glutaminase inhibitors (BPTES, CB-839) has shown higher in vitro anti-leukemic activities in IDH mutant cells than in IDH wild-type cells [28, 114], in line with the results obtained in gliomas [163]. However, although CB-839 clinical efficiency is currently being assessed in a phase 1 study in patients with AML (NCT02071927), in vivo preclinical studies have not been highly encouraging [27].

Interestingly, in IDH1 mutant glioma, 2-HG has been shown to inhibit branched-chain amino acid transaminases BCAT1 and BCAT2, which catalyze the degradation of BCAA into glutamate, increasing the dependency on glutamine to sustain glutamate and glutathione production and leading to synergy between glutaminase inhibition with CB-839 and radiation therapy [164, 165]. It would be particularly relevant to investigate BCAA in IDH mutant cells as Raffel et al. have already shown that BCAT1 mediates α-KG homeostasis in IDH WT AML and could represent a good therapeutic opportunity [166]. As demonstrated in gliomas, investigating the consequences of decreasing the BCAA pathway in IDH mutant AML and/or following treatments with IDH mutant inhibitors could pave the way toward a more efficient combinatory approach in myeloid leukemia. Furthermore, IDH mutation leads to higher mitochondrial activities in various solid cancers [162, 167–169], and the decreased NADPH levels associated with reduced wild-type activity in brain tumors and colorectal carcinomas [170–172] was partly restored by enhanced PPP activity in mutant astrocytes [173]. However, no detailed investigations of redox homeostasis in IDH mutant cells in AML have been reported to date, though Ward et al. suggested an increase in the activity of IDH wild-type enzyme may make a significant contribution to maintaining cellular and subcellular NADPH levels [140].

Key metabolic differences such as sensitivity to OxPHOS inhibitors seem to emerge in regard to cell lineage or cell types. Indeed, it has recently been reported that IDH1 mutant glioma cells were more resistant to rotenone (ETC complex I inhibitor) due to enhanced activity of pyrroline 5-carboxylate reductase 1 (PYCR1), which can oxidize NADH and produce proline as a ‘metabolic bypass’ of ETC complex I [174] (Fig. 1), while breast and colon cancer IDH1 mutant cells have been reported to be more sensitive to ETC complex I inhibition by metformin [167]. Of particular interest, overall response to a combination of venetoclax with azacitidine increased to 33% in IDH mutant subgroups of AML patients [15]. Chan et al. observed that (R)-2-HG inhibited cytochrome c oxidase activity (ETC complex IV), increasing the dependence on BCL2, and this led to higher sensitivity to ABT-199 in AML primary cells with an IDH mutation [175]. Notably, they observed a partial rescue of ABT-199 sensitivity with addition of specific IDH mutant inhibitors, which lower 2-HG levels [175].

### FMS-like tyrosine kinase 3 mutations

FMS-like tyrosine kinase 3 (FLT3) mutations, predominantly including internal tandem duplication defect (FLT3-ITD), are found in 30% of AML patients and confer a poor prognosis with enhanced relapse rate [176–179]. Clinical success of tyrosine kinase inhibitors (TKIs) against the oncogenic kinase BCR-ABL for CML treatment raised great expectations for FLT3 inhibitors in AML. However, although the initial response to monotherapy was promising (44% response in FLT3-ITD patients with relapsed/refractory AML treated with AC220, quizartinib [180, 181]), this did not result in prolonged disease-free survival [182]. The necessity to find new combinations has thus become apparent, underscoring the importance of better understanding FLT3-ITD specificities and linking this with inhibitor resistance (Table 1). Ju et al. first compared murine BaF3 cells with BaF3 cells overexpressing FLT3-ITD and observed enhanced glycolytic activity in FLT3-ITD cells, which was associated with higher phosphorylation of HK2 localized preferentially to mitochondria, favoring ATP transfer from OxPHOS to promote glycolysis. This also provides mitochondrial protection against mitochondrial death pathways by preventing opening of the mitochondrial permeability transition pore. Thus, a combination of glycolytic inhibitors with FLT3-ITD inhibitors produced encouraging results in vivo [24, 25], corroborating previous observations about 2-DG antileukemic activity in AML with FLT3-ITD or KIT mutations through glycosylation of oncogenic proteins [23].

Gregory et al. performed a synthetic lethality screen in AML cell line MOLM13 harboring a FLT3-ITD mutation and found that a number of the genes able to sensitize AML FLT3-ITD cells to FLT3 inhibitors were involved in metabolic processes [183], in particular the ataxia telangiectasia mutated (ATM) gene shown to activate G6PD to maintain redox homeostasis [184]. Furthermore, while AC220 treatment largely reverses the glycolytic phenotype, it also induces decreased glutathione metabolism, accumulation of mitochondrial ROS, and higher mitochondrial membrane potential, leading to an increased dependency on glutamine uptake to compensate. Thus, while not conveying benefit alone, AC220 efficacy in FLT3-ITD AML in vivo was increased by the addition of OxPHOS inhibitors or glutaminase inhibitors [51, 183, 185–187].

While described in many cancer types as a key deregulated metabolic pathway and promising therapeutic target [188–192], one-carbon metabolism in myeloid leukemia remains mostly unexplored. One carbon metabolism plays a crucial role in nucleotide synthesis, methylation processes, and redox homeostasis. Serine availability resulting from both increased uptake and de novo synthesis also appears to be a key player in tumorigenesis for various cancers [188, 193–195] but, to date, has not been reported in myeloid leukemia. However, Pikman et al. demonstrated that inhibition of methylenetetrahydrofolate dehydrogenase-cyclohydrolase 2 (MTHFD2) decreased AML growth, in particular in the FLT3-ITD subgroup [196]. MTHFD2 catalyzes the mitochondrial conversion of methylene-THF to formyl-THF using either NAD^+^ or NADP^+^ and is thus involved in purine biosynthesis, OxPHOS, redox homeostasis, and lipogenesis (Fig. 1).

Interestingly, an increasing number of studies focus on using current preclinical and clinical trials of these new drugs to better define their mechanisms of action and propose combinations with already FDA-approved treatments. In this context, the relevance of combining IDH mutant inhibitors with inhibition of oncogenic kinase signaling using TKIs has been demonstrated in two studies in AML [197, 198]. In one hand, Shih et al. have shown that combination of AC220 with the IDH2 mutant inhibitor AG-221 promotes better recovery of normal hematopoiesis and a reduction in mutant allele burden, targeting the mutant clone in vivo in Idh2^R140Q^Flt3^ITD^ AML mice [197]. On the other hand, Chen et al. recently pinpointed that both FLT3 WT and FLT3-ITD mutation increased the activity of IDH1 mutant AMLs through the activation of JAK2 by phosphorylation, providing a clinical rationale to combine FLT3 inhibitor and IDH1 mutant inhibitor regardless of FLT3 mutational status [198].

### Other AML patient mutational and cytogenetic subgroups

Surprisingly, metabolic dysregulation and/or specific biochemical characteristics are almost completely unknown in other karyotype and mutational patient subgroups with adverse risks, such as patients with p53, RAS, or CEBPα mutations, or monosomic complex karyotypes in AML. Notably, Fenouille et al. have shown that mitochondrial function was specifically driven by the creatine kinase pathway in the EVI1 subgroup of patients associated with poor prognosis [31]. EVI1 represses the myeloid differentiation regulator RUNX1, thus promoting expression of creatine kinase mitochondrial 1 (CKMT1). CKMT1 contributes to the conversion of arginine into creatinine. Pharmacological inactivation or genetic invalidation of CKMT1 abrogates ATP production and mitochondrial respiration, decreases viability of EVI1 AML, and prolongs the survival of the mice engrafted with high EVI1-expressing AML cells compared to xenograft with low EVI1-expressing AML cells. These observations highlight the therapeutic potential of targeting metabolic dependency specific to this EVI1 patient subgroup and show the necessity of identifying specific liabilities to achieve the best clinical outcome (Table 1).

## Current limitations in cancer metabolism studies and metabolism-based therapeutic strategies

Over the last 10 years, a number of increasing concerns emerged in cancer (metabolism) research about 1) reproducibility of published data [199–201], 2) differences of efficacy between in vitro and in vivo studies [93, 94, 202, 203] and 3) high attrition rates for cancer drugs [200, 204]. The models to use, the culture conditions, and the experimental design are undoubtedly at the heart of these discussions.

The example of striking discrepancies in anticancer efficacy of glutaminase inhibitor CB839 observed in vitro and in vivo highlights the crucial importance of tumor cell environment. Indeed, human non-small cell lung cancer (NSCLC) cells exhibit high sensitivity to CB839 treatment and displayed enhanced glutamine catabolism in vitro, while resistance to this inhibitor was observed in vivo [94]. Isotopic profiling experiments using ^13^C-glucose and/or ^13^C-glutamine performed in vivo in mouse KRAS-driven NSCLC and directly in patients using intraoperative ^13^C-glucose infusions revealed NSCLC tumors rely much more on glucose than on glutamine for TCA cycle anaplerosis in vivo [93, 94, 202]. Interestingly, Muir et al. cultured NSCLC cells in adult bovine serum, a medium in which component concentrations are much closer to in vivo models. In this culture medium, they observed that glutamine contribution to TCA was significantly lower compared to the classic in vitro conditions using fetal bovine serum, and thus comparable to in vivo data on glutamine metabolism and response to CB839 [203]. They went further, demonstrating that these differences relied on the level of a single nutrient, cystine (the oxidized dimer of the amino acid cysteine), present in classic in vitro conditions in concentrations 100-fold higher than in in vivo conditions. As the cystine level regulates glutamate export through the cystine/glutamate antiporter xCT, high levels of cystine in vitro lead to an increased export of intracellular glutamate and therefore a higher dependence on glutaminase activity to maintain glutamate level, and thus ultimately to enhanced sensitivity to CB839. Accordingly, these in vitro observations were not translatable to mouse and patient models [203]. These crucial studies highlighted the importance of taking into account how nutrient conditions can impact cell metabolism and response to therapies.

In this same vein, various efforts have been made to develop media with nutrient levels closer to those found in human serum such as human plasma-like medium (HPLM) [205] and Plasmax [206]. Cultures with these two media revealed that nutrient compositions of routinely used culture media can induce metabolic dependencies and rewiring that are not observed in vivo. One example of this is that growth of cancer and AML cells in HPLM containing human plasma levels of uric acid led to the inhibition of de novo pyrimidine synthesis. Indeed, uric acid is tenfold higher in human blood than in culture media and mice serum and can inhibit uridine monophosphate synthase (UMPS), and consequently reduces the sensitivity of cancer cells to the chemotherapeutic agent 5-fluorouracil [205]. Finally, large-scale RNAi and CRISPR screens are powerful tools to identify metabolic genes essential for cancer/AML cell proliferation and response to therapies. However, metabolic gene essentiality depends on cell culture medium, which is the major confounding factor affecting the reproducibility of such approaches [207]. This should especially be accounted for when investigating metabolic abnormalities in the context of tumor metabolic heterogeneity and to develop more effective metabolism-focused treatment strategies.

The above-mentioned studies indicate the importance of addressing metabolic reprogramming in the context of the microenvironment and developing combinatory therapeutic strategies. Directly linked to nutrient amounts and substrate availability in the niche, the notion of crosstalk between cancer cells and their neighbors should be taken into account. As we briefly mentioned in the previous section, MSCs and adipocytes have been shown to participate in and modulate the response to several therapies in AML, in particular through nutrient and metabolite releases or transfers. Co-cultures of AML cells with MSCs or with bone marrow adipocytes significantly reduced the sensitivity to CPT1a inhibitors [43, 208], reinforcing the major role of the microenvironment in sustaining energetic and anabolic demands. Notably, Tabe et al. reported that inhibition of CPT1a in AML increases free fatty acids and glucose uptake only in bone marrow adipocyte co-cultures, allowing blasts to preserve their viability [208].

A consideration of tumor metabolic systems biology is also allowing a better understanding of metabolic regulation, substrate utilization, and energy balance in whole organisms and will ultimately lead to better therapeutic strategies. Interestingly, AML cells were recently shown to hijack systemic glucose metabolism, inducing an insulin resistance with aberrant homeostasis in adipose tissues, pancreas, gut, and microbiota to desensitize normal tissues to glucose and support their own growth [209]. This study strongly suggests that 1) AML cells have a parasitic behavior in systemic host metabolism and that 2) organismal metabolic status is a key component of cancer/AML progression. Accordingly, recent studies have shown that nutrient availability from the environment/host, dietary regimens, and hormonal status can affect host insulin homeostasis and cancer cell metabolism to enhance drug efficacy [210, 211]. Moreover, non-cell autonomous autophagy, also called secretory autophagy (i.e., autophagy of cells in the tumor microenvironment), has recently been implicated in cancer metabolism by providing nutrients required to support anabolic cell growth and to satisfy cell demands in vivo for proliferation [212–214].

Finally, chemoresistance is the main cause of poor prognosis in AML patients and assessing the metabolic reprogramming of resistant LSCs after conventional chemotherapy or new treatments is an area of intensive research. A crucial point is that cells at relapse have been shown to be dramatically different in terms of phenotype or metabolism [48, 108, 215, 216]. Strikingly, Jones et al. have reported fundamental differences between therapy-naïve LSCs and LSCs at relapse [48]. As mentioned previously, they demonstrated that naïve LSCs are more dependent on amino acid uptake for OxPHOS maintenance and cannot up-regulate FA metabolism to preserve TCA cycle fueling in the absence of amino acids. However, they indicated that LSCs from relapse patients after conventional chemotherapy exhibit a new ability to compensate amino acid loss by enhancing FAO [48]. This could explain the decreased overall response to a combination of venetoclax with azacitidine in clinical trials for relapsed patients [217] compared to previously untreated patients [50]. Therefore, if de novo AML LSCs seem to be metabolically inflexible, at least regarding OxPHOS dependency, the ones resistant to chemotherapy and contributing to relapse are AML cells able to acquire metabolic flexibility and adapt [48, 218].

Altogether, these studies highlight the importance of better defining, better characterizing, and better designing our in vitro and preclinical studies as cell culture medium composition can significantly affect the response to metabolic pathway inhibition. Interestingly, comparisons between classic in vitro and more physiological medium also led to understanding some tumor metabolic specificities and dependencies and to propose new combinations of standard chemotherapeutic treatment or newly FDA-approved targeted therapies with metabolism-based drugs. Such studies should be conducted in AML and could, at least in part, explain the unsuccessful clinical translation of glutaminase or metabolic inhibitors, even though they displayed promising results in vitro and even in some studies in mice. These also show two major points in studying metabolic reprogramming to identify efficient clinical targets: 1) understanding metabolic cooperation, competition and symbiosis in the tumor microenvironment/niche is fundamental to tackling flexibility; and 2) primary tumor cell culture conditions impose critical experimental limitations to the study of cancer.

## Conclusion and perspectives

In the past decade, tremendous research efforts have uncovered key metabolic specificities and Achilles heels of cancer cells, including AML cells. These studies strongly suggest that myeloid leukemias are metabolic disorders and should be regarded in this light for metabolic-based personalized medicine treatments as well as for monitoring clinical responses to treatment. Several studies have further shown that AML cells, like other normal and cancer cells, are able to undergo compensatory metabolic and energetic adaptations in response to the inhibition of metabolic pathways, indicating that AML cells display complex metabolic capacities and flexibility that limit sustained drug efficacy, especially when challenged by chemotherapeutic drugs. However, targeting metabolic flexibility per se is not a feasible approach. By contrast, non-exclusive therapeutic strategies, which impede this metabolic flexibility by targeting its consequence(s), such as mitochondrial dependency, blocking the utilization of nutrients from the microenvironment, and/or targeting metabolic checkpoints, are emerging. Most of the metabolic pathways described in this review also occur in normal cells, although they are frequently less active, making the determination of the right therapeutic window difficult. Thus, if we are able to distinguish particular requirements of cancer cells to take up and utilize or eliminate certain metabolites, specifically targeting these exchanges may provide more effective treatment strategies. Finally, as already described in several solid tumors, an in vitro examination of metabolic flux networks does not reflect what occurs in situ, in vivo, and in patients due mainly to the enormous plasticity and heterogeneity of their metabolism [219, 220, 202]. AML, in common with many tumors, is highly genetically heterogeneous and its metabolism should be directly studied in patients in situ.

## Data Availability

Not applicable.

## Abbreviations

2-DG: 2-Deoxyglucose
2-HG: 2-Hydroxyglutarate
6PGD: 6-Phosphogluconate dehydrogenase
α-KG: α-Ketoglutarate
ACC2: Acetyl-CoA carboxylase 2
Akt: Protein kinase B
ALL: Acute lymphoblastic leukemia
AML: Acute myeloid leukemia
AMPK: 5' Adenosine monophosphate-activated protein kinase
ASNS: L-asparagine synthetase
ASS1: Arginosuccinate synthetase-1
ATM: Ataxia telangiectasia mutated
ATP: Adenosine triphosphate
BAK: Bcl-2 homologous antagonist/killer
BAX: Bcl-2-associated X protein
BCAA: Branched-chain amino acid
BCAT1/2: Branched-chain amino acid transaminases
BCL2: B-cell lymphoma 2
BH3: Bcl-2 homology domain 3
BRQ: Brequinar sodium
CKMT1: Creatine kinase mitochondrial 1
CML: Chronic myeloid leukemia
CN-AML: Cytogenetically normal AML
CPT1: Carnitine palmitoyltransferase 1
DHODH: Dihydro-orotate dehydrogenase
DHO: Dihydro-orotate
ETC: Electron transfer chain
EVI1: Ecotropic virus integration site 1 protein homolog
FADH_2_: Flavin adenine dinucleotide (hydroquinone form)
FAO: Fatty acid oxidation
FASN: Fatty acid synthase
FDA: Food and Drug Administration
FLT3: FMS-like tyrosine kinase 3
G6PD: Glucose-6-phosphate dehydrogenase
GBE1: Glycogen branching enzyme
GYS1/2: Glycogen synthase 1/2
HMG-CoA: 3-Hydroxy-3-methylglutaryl-coenzyme A
HPLM: Human plasma-like medium
IDH: Isocitrate dehydrogenase
JAK2: Janus kinase 2 non-receptor tyrosine kinase
LDHA: Lactate dehydrogenase A
LKB1: Liver kinase B1 protein
LSCs: Leukemic stem cell
MDS: Myelodysplastic syndrome
MSC: Mesenchymal stromal cell
mtDNA: Mitochondrial DNA
mTORC1: Mammalian target of rapamycin complex 1 protein
MTHF2: Methylenetetrahydrofolate dehydrogenase-cyclohydrolase 2
NADH: Nicotinamide adenine dinucleotide
NADPH: Nicotinamide adenine dinucleotide phosphate
NSCLC: Non-small cell lung cancer
OxPHOS: Oxidative phosphorylation
PC: Pyruvate carboxylase
PDH: Pyruvate dehydrogenase
PHD3: Prolyl-hydroxylase 3
PI3K: Phosphoinositide 3-kinase
PKM2: Pyruvate kinase PKM
PPP: Pentose phosphate pathway
PUMA: p53 upregulated modulator of apoptosis
PYCR1: Pyrroline 5-carboxylate reductase 1
ROS: Reactive oxygen species
SCD1: Stearoyl CoA desaturase 1
TCA: Tricarboxylic acid cycle or Krebs cycle
TK: Tyrosine kinase
TKI: Tyrosine kinase inhibitor
UMPS: Uridine monophosphate synthase
WT: Wild type
